# A gene expression atlas of the domestic pig

**DOI:** 10.1186/1741-7007-10-90

**Published:** 2012-11-15

**Authors:** Tom C Freeman, Alasdair Ivens, J Kenneth Baillie, Dario Beraldi, Mark W Barnett, David Dorward, Alison Downing, Lynsey Fairbairn, Ronan Kapetanovic, Sobia Raza, Andru Tomoiu, Ramiro Alberio, Chunlei Wu, Andrew I Su, Kim M Summers, Christopher K Tuggle, Alan L Archibald, David A Hume

**Affiliations:** 1 The Roslin Institute and Royal (Dick) School of Veterinary Studies, University of Edinburgh, Easter Bush, EH25 9PS, UK; 2 Fios Genomics Ltd, ETTC, King's Buildings, Edinburgh EH9 3JL UK; 3 Division of Animal Sciences, School of Biosciences, University of Nottingham, Sutton Bonington, Leicestershire LE12 5RD UK; 4 Department of Molecular and Experimental Medicine, The Scripps Research Institute, MEM-216, 10550 North Torrey Pines Road, La Jolla, CA 92037 USA; 5 Department of Animal Science, Iowa State University, Ames, IA 50011, USA; 6 Centre for Immunity, Infection and Evolution, University of Edinburgh Ashworth Labs, King's Buildings, West Mains Road, Edinburgh EH9 3JT; 7 Cancer Research UK, Cambridge Research Institute, Li Ka Shing Centre, Robinson way, Cambridge, CB2 0RE, UK

**Keywords:** pig, porcine, *Sus scrofa*, microarray, transcriptome, transcription network, pathway, gastrointestinal tract

## Abstract

**Background:**

This work describes the first genome-wide analysis of the transcriptional landscape of the pig. A new porcine Affymetrix expression array was designed in order to provide comprehensive coverage of the known pig transcriptome. The new array was used to generate a genome-wide expression atlas of pig tissues derived from 62 tissue/cell types. These data were subjected to network correlation analysis and clustering.

**Results:**

The analysis presented here provides a detailed functional clustering of the pig transcriptome where transcripts are grouped according to their expression pattern, so one can infer the function of an uncharacterized gene from the company it keeps and the locations in which it is expressed. We describe the overall transcriptional signatures present in the tissue atlas, where possible assigning those signatures to specific cell populations or pathways. In particular, we discuss the expression signatures associated with the gastrointestinal tract, an organ that was sampled at 15 sites along its length and whose biology in the pig is similar to human. We identify sets of genes that define specialized cellular compartments and region-specific digestive functions. Finally, we performed a network analysis of the transcription factors expressed in the gastrointestinal tract and demonstrate how they sub-divide into functional groups that may control cellular gastrointestinal development.

**Conclusions:**

As an important livestock animal with a physiology that is more similar than mouse to man, we provide a major new resource for understanding gene expression with respect to the known physiology of mammalian tissues and cells. The data and analyses are available on the websites http://biogps.org and http://www.macrophages.com/pig-atlas.

## Background

The comprehensive definition of the mammalian transcriptome has altered our view of genome complexity and the transcriptional landscape of tissues and cells. Systematic analysis of the transcriptome is of central interest to the biology community, but global coverage was not possible until the complete sequencing of the human and mouse genomes and the advent of microarrays. The pioneering work by Su *et al*. [1,2] provided the first comprehensive analysis of the protein-encoding transcriptome of major organs of human and mouse. Others have used microarrays or alternative methods to map expression in specific tissues or cell types [3-7]. The work of the FANTOM and ENCODE projects has revealed the true complexity of the mammalian transcriptome, highlighting the impact of alternative initiation, termination and splicing on the proteome, and the prevalence of multiple different classes of non-coding RNAs (ncRNAs) [8-11]. The pace of data acquisition has continued to grow with the increasing reliability and decreasing cost of the core technologies such as microarrays and the sequencing of RNA (RNAseq). Despite these efforts, knowledge of the human transcriptional landscape is still sparse. Efforts to curate and analyze an 'atlas' from the existing human microarray data are hindered by the fact that certain types of samples have been analyzed extensively, for example hematopoietic cells and cancers, while little or no data are available for many other tissues and cell types [12]. Studies of the non-pathological human transcriptome are compromised further because most tissues can only be obtained post-mortem, the provenance of samples can be variable and the health status of the individual from whom they were obtained is often unknown.

With numerous predicted mammalian protein-coding loci still having no informative functional annotation and even less insight into the function of the many non-protein-coding genes, detailed knowledge of a transcript's expression pattern can provide a valuable window on its function. Previously, we have used coexpression analysis of large mouse datasets to provide functional annotation of genes, characterization of cell types and discovery of candidate disease genes [13-16]. Isolated cell types may differ not only in their specialized function but also in their engagement with 'housekeeping' processes, such as growth and proliferation, mitochondrial biogenesis and oxidative phosphorylation, metabolism and macromolecule synthesis, the cytoskeleton, the proteasome complex, endocytosis and phagocytosis. Genes coding for proteins within pathways, both generic and cell-specific, often form coexpression clusters [14], so one can infer the function of a gene of unknown function from the transcriptional company it keeps, by applying the principle of guilt-by-association. The identification of coexpression clusters can, in turn, inform the identification of candidate genes within genomic intervals associated with specific traits from genome-wide association studies (GWAS) or classical linkage studies. For example, we identified a robust cluster of genes that is expressed specifically in cells of mesenchymal lineages in the mouse [14-16]. The cluster contained a large number of genes previously shown to be causally associated with inherited abnormalities of the musculoskeletal system in humans [14-16]. By inference, other genes within this cluster that have less informative annotation or no known function, are likely to be involved in musculoskeletal development. As noted previously [17], the conservation of coexpression clusters can provide an even more powerful indicator of likely conserved function. These authors mapped coexpressed clusters onto 850 human Mendelian disease loci of unknown molecular basis from Online Mendelian Inheritance in Man (OMIM) and identified 81 candidate genes based upon their conserved restricted expression within the affected organ.

The domestic pig (*Sus scrofa*) is economically important in its own right, and has also been used increasingly as an alternative model for studying human health and disease and for testing new surgical (including transplantation) and pharmacological treatments (reviewed in [18,19]). Compared to traditional rodent models, the pig is more closely-related to humans in its size, growth, development, immunity and physiology as well as its genome sequence [20]. The translation of preclinical studies in rodents into clinical applications in humans is frequently unsuccessful, especially for structures where rodents have very different anatomy and physiology, such as the cardiovascular system [21,22]. The recently released pig genome sequence (Sscrofa10.2, ftp://ftp.ncbi.nih.gov/genbank/genomes/Eukaryotes/vertebrates_mammals/Sus_scrofa/Sscrofa10.2/) [23] and associated annotation will greatly enhance the utility of the pig as a model [24]. However, compared to the mouse, the knowledge of the pig transcriptome is very limited partly due to a lack of commercial expression microarrays with comprehensive gene coverage [25]. While several EST (Expressed Sequence Tag) sequencing projects have explored gene expression across a range of tissues [26-28], a systematic global study of the tissue expression landscape is not available. Here we present a new microarray platform for the pig with greatly improved gene coverage and annotation. We have used this array to generate an expression atlas for the pig, comparable to the human/mouse expression atlases, and, using advanced visualization and clustering analysis techniques, we have identified networks of co-expressed genes. A detailed analysis of the porcine gastrointestinal tract illustrates the power of the analytical approach and data. These data will support improved annotation of the pig and human genomes and increase the utility of the pig as a model in medical research.

## Results and discussion

The pig is uniquely important both as a major source of food and an animal model for human disease. Until recently the lack of a genome sequence for the pig and consequently many of the functional-genomic analysis tools, have limited the kind of analyses now routine in human and mouse systems. Here we report the design, annotation and validation of a new comprehensive microarray for the analysis of gene expression in the pig and a first attempt to produce a global map of the porcine protein coding transcriptome.

The new Snowball array (named after the Trotsky pig character in George Orwell's novel *Animal Farm *[29]) is far more comprehensive in its gene coverage than the previous porcine Affymetrix array which was based on the available expressed sequence tag data circa 2004. It is also more extensive than the new porcine 'peg' array (PorGene-1_0-st-v1) recently released by Affymetrix (Table 1), with nearly twice as many probes included on the Snowball array, and draws on a larger cDNA sequence database. The results from the analysis described here validate the performance and gene annotation of the Snowball array. A major problem currently restricting genomic analysis of production animals is the fact that many genes remain unannotated due to problems in establishing orthology among homologous sequences from other species. We adopted a 'best match' approach to increase the number of annotated features on the array. The repeated finding that transcripts annotated in this way were expressed in a pattern that was consistent with their proposed function (where known) supports the validity of this approach. However, we would urge caution in accepting the orthology match of probes annotated in this way without further verification. We have aligned the probe sequences from the Snowball array with the recently released Sscrofa10.2 assembly. We will publish these alignments as a DAS track in Ensembl in the short term and integrate the alignments into Ensembl and Biomart in the next Ensembl release. These alignments enable the expression data to be used to annotate the genome sequence further and the interpretation of expression profiles for a gene/transcript in a genomic context.

**Table 1:** Comparison of Affymetrix arrays designed for analysis of the pig transcriptome.

	Porcine Genome Array	PorGene-1_0-st-v1	Snowball
	
Genome build	UniGene Build 28 (2004)	Genome build 9	Genome build 9
Design type	3' bias	whole transcript	whole transcript
Number of probes	531,272	572,667	1,091,987
Number of probesets	23,937	144,644	47,485
Mismatch probes	Yes	No	No
Includes non-coding RNAs	No	No	Yes

A comparison of different porcine expression arrays manufactured by Affymetrix. This table compares the major features of the two commercial Affymetrix expression arrays that have been designed for studies of the pig transcriptome with the array designed and used for this study.

Arrays still provide a very cost effective solution for producing a large amount of high quality gene expression data. In terms of speed of data acquisition and availability of established analysis routines that can be run on desktop machines, arrays still have many advantages over sequencing-based analyses. With improvements in the assembly and annotation of the genome and gene models and RNAseq analyses increasing our knowledge of the transcriptional landscape of the transcriptome, there is no doubt the current array design will be enhanced.

The primary cohort of animals used for this study was a group of three- to four-month old juvenile pigs of both sexes. We aimed to gather samples of every major pig tissue. Where possible biological replicates were analyzed that originated from different animals of each sex. Regional analysis of the brain is clearly important, and more feasible in pigs than in mice, but the method of killing (cranial bolt) meant that detailed dissection of brain was not possible. The age/stage of the animals also meant that certain tissues could not be collected and the panel of tissues was supplemented by samples of placenta and a mature testis (since these are major sites of tissue restricted gene expression) [1,2]. Since macrophages have proved to be one of the most complex sources of novel mRNAs [9], we included a number of macrophage samples (with or without lipopolysaccharide (LPS) stimulation) in the atlas. For details of the tissues and cells used for this study see Additional file 1, Table S1.

BioLayout *Express*^3D ^[30,31] is a unique tool in the analysis of large complex expression datasets. The statistical approach employed centers on the principle of coexpression, based on the transcript-to-transcript comparison of the expression signal across the samples analyzed, by calculation of a Pearson correlation matrix. For any given comparison, the Pearson value can range from +1 (perfect correlation) to -1 (perfect anti-correlation). The correlation and clustering algorithms within BioLayout *Express*^3D^, together with the ability to visualize and explore very large network graphs, mean that it is uniquely positioned for the analysis of large datasets and has been used extensively for this purpose [14,16,32-34]. A graph derived from a given correlation cut-off value includes only those genes that are related in expression to others above the selected threshold and more or less complex graphs may be analyzed by decreasing or increasing this value, respectively. Core topological structures that often form separate graph components at high thresholds are robust and are maintained as correlation cut-off values are lowered.

**Figure 1:**
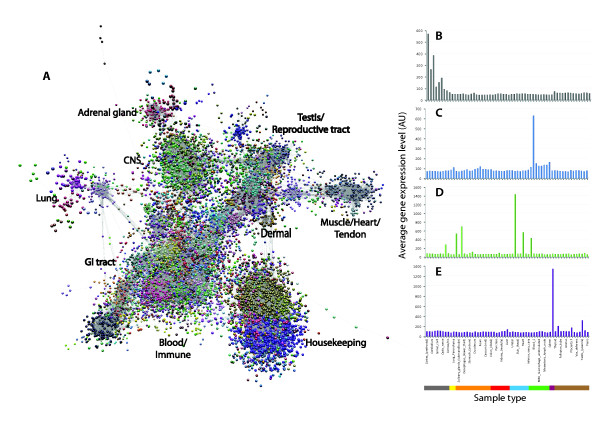
**Network visualization and clustering of the pig transcriptome**. **A**. Three-dimensional visualization of a Pearson correlation graph of data derived from analysis of pig tissues and cells. Each node (sphere) in the graph represents an individual probeset on the array and the edges (lines) correspond to correlations between individual measurements above the defined threshold. The graph is comprised of 20,355 nodes (probesets) and 1,251,575 edges (correlations ≥0.8). The complex topology of the graph is a result of groups of co-expressed genes forming cliques of high connectivity within the graph. Clustering of the graph using the MCL algorithm was used to assign genes to groups based on coexpression. By inspection of the underlying profiles, areas of the graph can be associated with genes expressed by specific tissue or cell populations. Plots of the average expression profile of genes in selected clusters are given on the right: **B**. profile of cluster 4 genes whose expression is restricted to brain and spinal cord; **C**. profile of cluster 7 genes whose expression is highest in blood; **D**. profile of cluster 10 genes whose expression is restricted to skeletal muscle; **E**. profile of cluster 22 genes whose expression is highest in the adrenal gland. MCL, Markov cluster algorithm.

**Figure 2:**
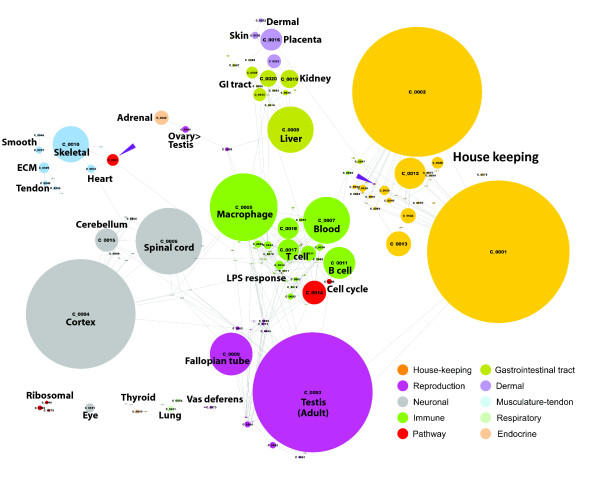
**Network topology of porcine expression atlas**. The collapsed cluster diagram shown here is a simplified view of the graph used for this analysis and shown in Figure 1. Each node represents one of the 150 largest clusters of genes, the size of the node being proportional to the number of individual nodes (probesets) within that cluster. Edges represent connections between clusters whereby nodes in one cluster share edges with nodes in another. The color of the nodes has been selected to represent clusters of genes expressed in given types of tissues which tend to group together with the overall topology of the network.

We used BioLayout *Express*^3D ^to analyze the pig transcriptome data generated using the Snowball array (all normalized expression data is provided in Additional file 2). From a pairwise transcript-to-transcript correlation matrix a weighted, undirected network graph was constructed using a Pearson correlation threshold cut-off of r ≥ 0.80. The resultant graph was large and highly structured (Figure 1, Additional file 3) with one large component of 19,708 nodes and 90 smaller components (unconnected networks of correlations) of between 57 and 5 nodes (20,352 probesets in total, that is, just under half the transcripts represented on the array). The topology of the graph contained localized areas of high connectivity and high correlation (representing groups of genes with similar profiles), dominated by groups of genes that are coexpressed and form highly connected cliques within the network (Figures 1 and 2). Nodes representing different probesets designed to the same gene were generally highly correlated and connected to each other in the graph, confirming the validity of the probeset annotation and approach.

**Figure 3:**
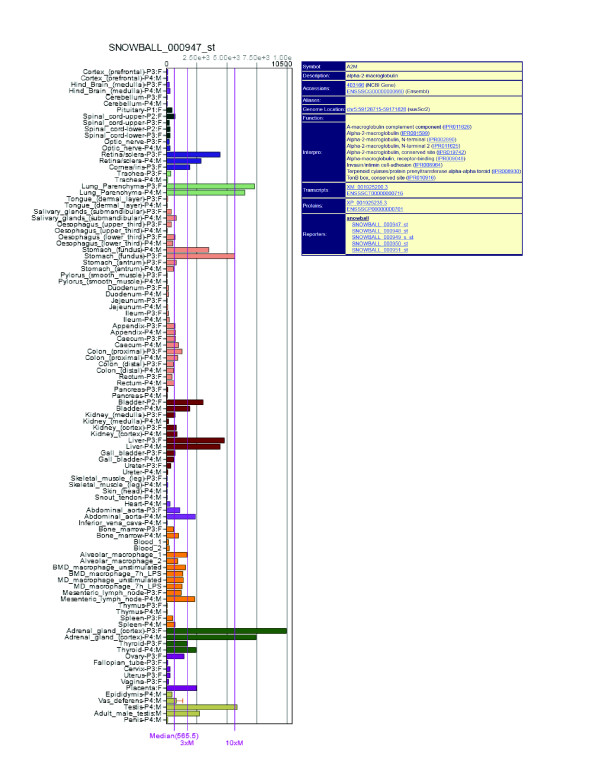
**Screenshot of the representation of the profile of the pig *A2M *gene within the BioGPS online portal**. All data used for this study are available through the BioGPS database. Genes can be searched for and where found the full expression profile is displayed as a bar chart. Samples are colored according to type, for example, CNS, GI tract, and so on, and the interface supports a number of useful features including a zoom function on the profile viewer, searches for genes with a similar profile, access to the raw data, links to external resources and the potential to compare profiles across species, for example, human, mouse, rat, zebrafish, frog. CNS, central nervous system; GI, gastrointestinal.

Some highly expressed genes were not included in the graph. The more unique a gene's expression pattern, the fewer neighbors it will have in the network. One example is the protease inhibitor, alpha-2-macroglobulin (*A2M*). There were five probesets on the array designed to this gene and all showed a highly similar expression pattern, albeit at a range of signal intensities. These probesets formed a small correlation network with themselves, but the expression pattern of this gene in the context of the full atlas was essentially unique and no other porcine gene was expressed in this manner (Figure 3). In some cases, such isolation is a consequence of the use of distinct cell-restricted promoters [10,32]. For *A2M*, there is a single major transcription start site in both mouse and human, and the pattern of expression is similar in these two species ([10]http://biogps.org) and in pig, suggesting that a common set of regulatory factors control this gene's expression across species. For the majority of other probesets not found in the graph described here, transcripts appear to be expressed at very low levels (or not at all). These genes may be highly-expressed in cells or tissues we have not sampled in this sample set. For example, we would not detect genes exclusively expressed during prenatal life as no samples from these stages were represented in the current atlas.

Clustering of the graph using the Markov clustering algorithm (MCL; see Materials and Methods) resulted in 1,945 clusters (n >1). The largest consisted of 1,308 transcripts and the top 153 clusters (consisting of ≥10 probesets), accounted for 68.6% of the nodes in the graph. The remainder of the graph was of a sparser topology and subdivided into numerous small clusters. Figure 1 shows the overall topology of the network graph together with the expression profiles of selected clusters. The profile and gene content of each cluster was examined in detail, and the 50 largest clusters are shown in Table 2. The full cluster list together with gene membership is supplied in Additional file 4, Table S2. Note that there may be a degree of variation in the expression pattern of individual genes within a cluster which is masked when average profiles are displayed.

**Table 2:** List of 50 largest network clusters and association with particular tissue/cells/pathway.

Cluster ID	Number of transcripts	Profile Description	Class	Sub-class
5	622	Macs-other immune	Immune	Macrophage

18	195	Alveolar macs>>other macs	Immune	Tissue macrophage

32	81	LPS-induced (high in other tissues)	Immune	Immune response (IFN)

35	67	LPS-induced mac-specific	Immune	Immune response (LPS)

34	73	MHC class1-related	Immune	MHC class I

11	293	Thymus>Blood>spleen	Immune	T cell

38	65	Small intestine (jej./ileum)>blood-spleen-LN	Immune	B cell

7	430	Blood>>macs-other immune	Immune	Blood

17	197	Blood>macs>spleen (immune)	Immune	Blood

21	139	Blood-immune organs>GI tract (immune)	Immune	Blood

4	1007	CNS-highest in cortex	CNS	Neuronal

15	213	Cerebellum>>other CNS	CNS	Cerebellum

6	611	CNS-high in spinal cord	CNS	Astrocyte

41	59	Retina	CNS	Retinal epithelium

8	425	Liver (hepatocyte)	GI tract	Liver

16	201	Tongue Esophagus>skin	GI tract	Stratified epithelium

19	168	Kidney cortex>medulla-liver	GI tract	Kidney epithelium

20	155	Small intestine>>kidney-liver	GI tract	SI epithelium (enterocyte)

25	121	GI tract>>gall bladder	GI tract	Epithelial

26	110	GI tract>>others	GI tract	Columnar epithelial

45	53	Pancreas	GI tract	Exocrine pancreas

46	51	Liver>kidney	GI tract	Hepatocyte

47	49	Salivary gland	GI tract	Salivary gland acinar cell

10	333	Skeletal muscle>heart	Musculature-tendon	Musculature

29	98	General-low in macs/CNS	Musculature-tendon	Fibroblast (ECM)

33	75	Heart>upper oesophagus	Musculature-tendon	Musculature

37	66	Smooth muscle (high in many)	Musculature-tendon	Musculature

49	46	Snout tendon>trachea	Musculature-tendon	Cartilage-tendon

23	128	Placenta	Dermal	Placental function

36	67	Skin>>tongue	Dermal	Dermal

22	131	Adrenal gland>>>ovary-placenta-testis	Endocrine	Steroid hormone biosynthesis

31	84	Thyroid gland	Endocrine	Thyroxine biosynthesis

3	1102	Testis-adult	Reproduction	Gamete production

42	59	Testis-adult	Reproduction	Gamete production

9	392	Fallopian tube>adult testis	Reproduction	Female

40	61	Ovary>Testis (juvenile)	Reproduction	Female

44	56	Testis>other	Reproduction	Cell cycle-related

14	218	Many tissues-highly variable	Pathway	Cell cycle

27	108	General but not even	Pathway	Oxidative phosphorylation

48	48	General but not even	Pathway	Histones

50	43	General but not even-highly expressed	Pathway	Ribosomal

24	124	General but not even	House keeping	House keeping

28	108	General but not even	House keeping	House keeping

43	57	General but not even	House keeping	House keeping

1	1309	General, relatively even	House keeping	House keeping (HK1)

2	1193	General, relatively even-low in macs	House keeping	House keeping (HK2)

12	287	General, relatively even	House keeping	House keeping (HK3)

30	87	General, relatively even	House keeping	House keeping (HK4)

39	65	General, relatively even	House keeping	House keeping (HK5)

13	229	Spinal cord 1 rep only (tech artefact)	Tech artefact	

A list of the 50 largest coexpression clusters evident in the pig gene expression atlas. Listed in the table are the 50 largest clusters of genes originating from our analysis of the pig expression atlas. Clusters are numbered according to their size (the largest being designated cluster 1) but are sorted here according to their grouping within the graph and the biology they represent. The first two columns give the cluster ID and number of transcripts present in the cluster and the following column provides a description of the average expression profile of all transcripts within the cluster. The final two columns aim to group clusters according to the class of tissues in which these genes are predominately expressed and the tissue, cell or pathway we believe they represent.

Several of the largest clusters showed relatively little tissue specificity in their expression and might be considered to be 'housekeeping' genes since the proteins they encode are likely to be functional in all cell types. Such clusters are a common feature of large correlation graphs where a relatively low threshold has been employed. Genes/probes with limited informative nomenclature were over-represented in these clusters, perhaps reflecting previous research focus on genes that demonstrate tissue-restricted expression profiles [32]. Aside from these large, nondescript clusters, the majority of the coexpression clusters were made up of transcripts that have a distinct tissue/cell restricted expression pattern. In each case, the cluster was named based upon the tissue/cell(s) in which the genes were most highly-expressed. These data recapitulate many of the known tissue restricted expression patterns that have been described for human and mouse [1,2]. For example, there were multiple large clusters of genes with strong expression in the macrophage samples with a subset more highly-expressed in the alveolar macrophages and another set induced by LPS. Each of these clusters contained genes for numerous well-studied macrophage surface markers and receptors, and proinflammatory cytokines. A detailed comparative analysis of human and pig macrophage gene expression has been reported elsewhere [33]. The present analysis did not identify the single large phagocytosis/lysosome functional cluster that was evident in the analysis of mouse primary cell data [14,32]. This cluster tends to be broken up when tissue samples are included in the analysis because many of the components of this system are utilized more generally in vesicle-trafficking and in other pathways.

A secondary feature of the network graph is that clusters with similar expression patterns formed neighborhoods (Figure 2). For instance, clusters of genes selectively expressed in the reproductive tract, gastrointestinal tract, central nervous system (CNS), mesenchymal-derived tissues, dermal tissues or blood cells tended to occupy similar areas. In this way the graph distributed the transcriptome into groups of genes associated with tissues composed of cells of different embryonic lineages.

Because cells and tissues differ in their engagement with fundamental biochemical processes, the graph also contained clusters that grouped together genes associated with a particular cellular process (pathway) which may be active in a wide range of tissues albeit not at the exact same level. Examples include clusters enriched for ribosomal (clusters 50, 65, 79 and 184), cell cycle (cluster 14) and oxidative phosphorylation (clusters 27 and 99) genes. The clusters of ribosomal genes form a separate graph component which together contain 106 transcripts (approximately 94 genes), including at least 37 known ribosomal protein genes (others appear in the list but are annotated with LocusLink (LOC) gene identifiers), genes for eukaryotic translation initiation factors (EEF1B2, EIF3E, EIF3H), two members of the RNaseP complex, NACA (nascent polypeptide-associated complex alpha subunit), U1 and U4 small nuclear ribonucleoproteins and at least 23 small nucleolar RNAs (snoRNAs). snoRNAs function to guide modifications of other RNAs, particularly ribosomal protein mRNAs [35], consistent with their co-clustering with components of the ribosome complex. Different tissues also vary in their rates of cell renewal and consequently in the proportions of proliferating cells. Genes involved in the cell cycle, therefore, have a pattern of expression that reflects the mitotic activity of the tissues and such genes are readily identified in the graph. Cluster 14 contains many genes for proteins known to be involved in the cell cycle (GO term enrichment analysis of this cluster returned *P*-values of 5.2 × 10^-60 ^for 'cell cycle' and 2.9 × 10^-51 ^for 'mitosis') and supports the involvement of other cluster 14 genes in this pathway. For example, the cluster includes vaccinia-related kinase 1 (*VRK1*) shown recently to play a role in the control of mitosis [36], highlighting the importance of our approach for annotation of uncharacterized genes.

To further illustrate the power of this approach in defining pathway systems, we show a detailed analysis of the enrichment of genes associated with oxidative phosphorylation and the tricarboxylic acid (TCA) cycle in clusters 27 and 99 (Table 3). Clusters 27 and 99 were widely separated within the graph (see Figure 2). This separation represents a different regulation of these two sets of genes. All cluster 99 genes (17 transcripts) were highly expressed in all tissues (hence their close association with the housekeeping clusters) and are core components of the mitochondrial oxidative phosphorylation complexes encoded by the mitochondrial genome. In contrast, the genes in cluster 27 are encoded by the nuclear genome and showed a marked elevation in their expression in the heart, reflecting the high rates of respiration in this tissue. The 108 transcripts in this cluster include multiple members of every one of the five complexes associated with the generation of ATP by the mitochondria and most of the enzymes driving the TCA cycle. The coexpression of multiple members of pathways for long chain fatty acid oxidation, mitochondrial membrane transport and ubiquinone and cytochrome C biosynthesis supports the functional link between these pathways [37,38]. On the basis of guilt-by-association the unnannotated/poorly characterized transcripts within this cluster are prime candidates for a functional association with the oxidative respiration process. For example, *GBAS *and *CHCHD10 *were recently identified by coexpression analysis and shown to be associated with mitochondrial complex IV [39]. There are numerous other clusters within this dataset which cannot easily be associated with an obvious functional role but likely represent clusters of genes with shared or related functions.

**Table 3:** Genes associated with the oxidative phosphorylation pathway present in clusters 27 and 99.

Functional Grouping	Cluster 27	Cluster 99
TCA cycle	*ACO2, CS, FH, IDH2, IDH3B, MDH2, SUCLG1*	
Oxidative phoshorylation, Complex I	*NDUFA1, NDUFA10, NDUFA12, NDUFA8, NDUFA9, NDUFAB1, NDUFB1, NDUFB2, NDUFB3, NDUFB5, NDUFB6, NDUFB8, NDUFB9, NDUFC1, NDUFC2, NDUFS1, NDUFS2, NDUFS6, NDUFV2, NDUFV3*	*MT-ND1, MT-ND2, MT-ND3, MT-ND4, MT-ND4L*, *MT-ND5*,
Oxidative phoshorylation, Complex II	*SDHA, SDHB*	
Oxidative phoshorylation, Complex III	*CYC1, UQCR10, UQCRB, UQCRC1, UQCRFS1, UQCRH*	*MT-CYB*
Oxidative phoshorylation, Complex IV	*COX4I1, COX5B, COX6B, COX6C, COX7B2*	*MT-CO1, MT-CO2, MT-CO3*
Oxidative phoshorylation, Complex V	*ATP5A1, ATP5C1, ATP5F1, ATP5G1, ATP5G3, ATP5J2, ATP5H*	*MT-ATP6*
Cytochrome C biosynthesis	*HCCS*	
Fatty acid (long chain) beta-oxidation	*ACADVL, GOT2, HADHA, HADHB, PTGES2*	
Mitochondrial membrane transport	*CHCHD3, NNT, SAMM50, TIMM8B, TOMM7, TUFM, VDAC1*	
Mitochondrial RNA processing	*SLIRP, MRPL2, MRPS24*	
Ubiquinone biosynthesis	*COQ6, COQ7, COQ9*	
Apoptosis-associated	*AIFM1, DELE*	
Ox phos-related	*BOLA3, BRP44, CHCHD10, GBAS*	
Unknown function	*C11orf67, C6H4orf52, IMMT, LOC100060661, LOC100512781, LOC100520866, LOC100523804, SS18L2, WDR45*	*Gm8437, LOC100512762, LRP1B, MTRNR2L4*

Coexpression of members of oxidative phosphorylation and related pathways. Nuclear (cluster 27) and mitochondrial (cluster 99) gene clusters associated with oxidative phosphorylation. The known association with pathway/protein complex is listed for each gene in the cluster. List of all genes present in clusters 27 and 99 and the function of the encoded protein, where known.

**Figure 4:**
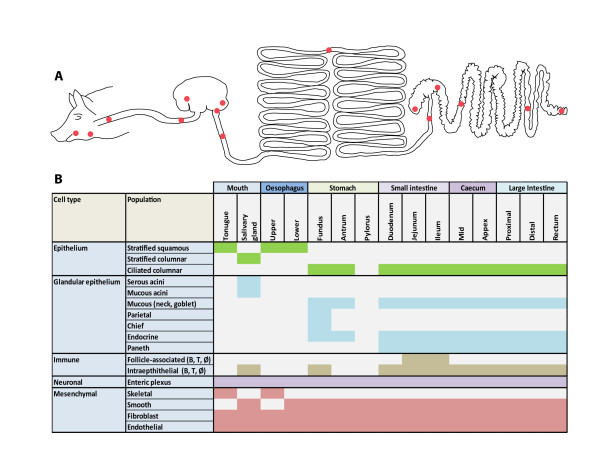
**Diagram of pig GI tract and table of the cell populations/structures associated with specific regions**. **A**. Schematic of the different regions of the pig GI tract with areas sampled for this study marked with a red dot. **B**. Table of the five main cell types and subdivisions thereof that make up the GI tract and their expected presence in the samples analyzed here. GI, gastrointestinal.

The pig's size and the feasibility of obtaining fresh tissues from healthy individuals offer a unique opportunity to study the expression landscape of important organ systems. In common with humans, the pig is an omnivore and its gastrointestinal tract (GI) has evolved to be able to masticate, digest and absorb a wide range of foodstuffs. In this study, we collected samples along the entire length of the GI tract from the tongue to the rectum, a total of 15 distinct regions (in duplicate), as shown in Figure 4a. The GI tract is lined with an epithelial layer whose cellular composition changes in line with the functional role of the GI compartment. The upper GI tract is lined with a stratified squamous epithelium which transitions in the stomach to a columnar epithelium that runs through to the rectum. Even within the small intestine, enterocyte expression of solute transporters and digestive enzymes is tightly regulated to reflect the changing nature of the luminal contents, as well as the migration of cells up the crypt-villus axis [40]. Associated with the epithelium are various glandular cell types involved with enzyme secretion, lubrication, and endocrine control, and specialized structures, such as the pyloric and fundic glands of the stomach and sub-mucosal Brunner's glands of the duodenum. The lamina propria, which lies beneath the epithelium, is itself a complex mix of cells made up of endothelial, immune and connective tissues. The GI tract is almost entirely surrounded by musculature (predominately smooth muscle) and regulated by the enteric neural plexus. Therefore, the GI tract is composed of five major classes of cell types: epithelia, glandular/endocrine epithelia, immune cells, neuronal cells and mesenchymal cells (muscle, connective tissue). The region-specific cellular composition of the GI tract is summarized in Figure 4b.

To validate the GI-specific analysis, we initially selected a number of gene families/classes where expression is known to be specific to certain cell populations in other mammals [see Additional file 5, Figure S1]. Keratins are structural proteins that distinguish different classes of epithelial cells [41]. We looked at eight keratin gene family members (Figure S1a). All but *KRT8 *and *KRT19 *were heavily expressed in the tongue, *KRT5, KRT13 *and *KRT78 *were also expressed in the lower esophagus and fundus, both of which are lined with a stratified squamous epithelium. *KRT8 *and *KRT19*, markers of columnar epithelium [42,43], showed the anticipated inverse pattern, with strong expression in the salivary gland, antrum and along the entire length of the small and large intestine. To confirm region-specific epithelial function, we examined the expression of four well-characterized brush border hydrolases: lactase (*LCT*), sucrose-isomaltase (*SI*), aminopeptidase N (*ANPEP*) and dipeptidyl-peptidase 4 (*DPP4*) (Figure S1b). *LCT *is responsible for the enzymatic cleavage of the milk sugar lactose and was detected in the duodenum and jejunum but not in the ileum. *SI *expression was low in the duodenum and peaked in jejunum, with lower expression in the ileum. *ANPEP *and *DPP4 *were expressed all along the small intestine. *DPP4 *was also highly expressed in the salivary gland and in the distal colon. These observations fit the known expression patterns for these genes in post-weaned rabbits [40]. Associated with the role of the intestine in nutrient uptake, there were a large number of solute transporters included in the GI tract data (86 members of the SLC family alone), and many showed region-specific expression patterns consistent with their known functions (Figure S1c). For example, ferroportin (*SLC40A1*), a protein involved in iron export from duodenal epithelial cells and found to be defective in patients with iron overload [44,45], was restricted to duodenum. The expression of the enterocyte sodium/glucose cotransporter (*SLC5A1*) was restricted to the small intestine, expression levels peaking in the jejunum [46] and the chloride transporter of apical membrane of columnar epithelium of the colon (*SLC26A3*) [47] which when mutated results in congenital chloride diarrhea, was largely restricted to the large bowel samples. Other cell-specific 'marker' genes, for example, mucins (salivary gland: *MUC12, MUC19*; stomach: *MUC1, MU5AC*; colon: *MUC4*), gut hormones (stomach: *GKN1, GKN2*; duodenum: *CCK, GKN3, MLN*), lymphocyte markers (T cell: *CD2, CD3D/E, CD8A*; B cell: *CD19, CD22, CD79A/B, CD86*), myosins (smooth muscle: *MYL6, MYL9*; skeletal muscle: *MYL1, MYL3, MYL4*) and collagens (connective tissue: *COL1A1, COL1A2, COL5A1, COL6A1*) were also enriched in samples where they would be expected (Figures S1d-h, respectively).

**Figure 5:**
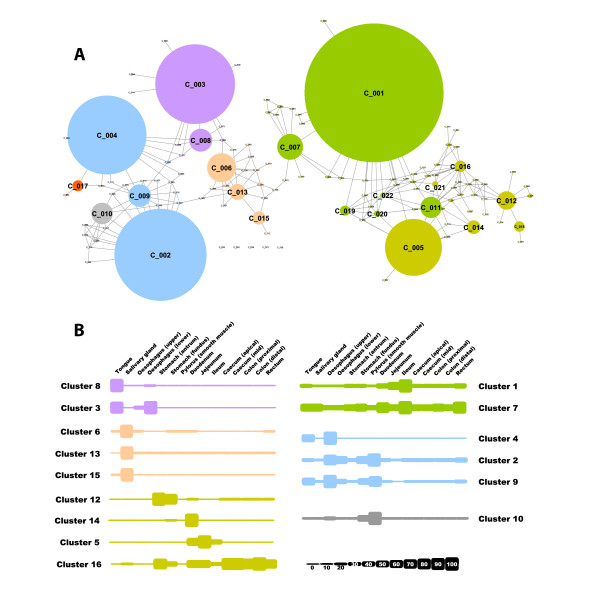
**Collapsed cluster diagram of porcine GI tract expression network together with the average gene profile of transcripts within selected clusters**. **A**. Collapsed cluster diagram shown here is a simplified view of the graph used for the analysis of the GI tract [see Additional file 6, Figure S2 for screenshot of transcript level graph]. Each node represents a cluster of genes, the size of the node being proportional to the number of individual nodes (probesets) with that cluster. Edges represent connections between clusters whereby nodes in one cluster share edges with nodes in another. The color of the nodes has been selected to represent clusters of genes expressed in similar tissue types. **B**. Block diagrams of the average gene expression profile of the major GI clusters. Expression levels are indicated as a % of maximum with values rounded to the nearest 10%, each GI compartment analyzed being represented as a separate block. A key to the size of each block is shown in the bottom right hand corner. Gene clusters have been grouped according to cell type of origin: purple, stratified squamous epithelia; brown, salivary stratified columnar epithelia; light green, ciliated/glandular columnar epithelia; dark green, immune cells/cell cycle; blue, musculature (smooth and skeletal); grey, neuronal. GI, gastrointestinal.

The GI tract data were prefiltered to remove low intensity signals and technical artefacts, and the remaining data (from 5,199 probesets) subjected to network analysis. A collapsed cluster diagram of the network is shown in Figure 5a and screenshots of the transcript level network in Additional file 6, Figure S2. Annotated '.expression' and '.layout' files are given in Additional files 7 and 8, respectively. The data divided into 120 clusters of coexpressed genes (Figure 5b). A listing of the main clusters and an interpretation of the gene signatures is shown in Table 4 and a full listing of the genes within those clusters is provided in Additional file 9, Table S3.

**Table 4:** Cluster analysis summary of transcripts expressed in a region-specific manner along the porcine GI tract.

GI clusters r = 0.9, MCL1.7	Probes	Unique gene IDs	Cluster Expression Profile Description	GI-cluster: Class	GI-cluster: Sub-Class
Cluster003	460	372	Tongue/lower_esophagus	Stratified squamous epithelium	Epithelium
Cluster008	132	114	Tongue>>lower_esophagus	Stratified squamous epithelium	Epithelium
Cluster016	66	52	Stomach/intestine	Columnar epithelial	Epithelium
Cluster005	328	251	Small intestine	Digestion/absorption	Epithelium
Cluster028	21	17	General-higher in fundus/intestine	Epithelial	Epithelium
Cluster021	35	29	Intestine	Intestinal epithelium	Epithelium
Cluster013	93	76	Salivary gland	Stratified columnar epithelium	Epithelium
Cluster025	23	19	Colon specific	Colon epithelial specific function	Epithelium
Cluster029	21	18	Colon>>fundus	Colon epithelial specific function	Epithelium
Cluster040	16	8	Colon>>fundus/small intestine	Colon epithelial specific function	Epithelium
Cluster015	76	65	Salivary gland	Mucous acini	Glandular epithelium
Cluster006	167	135	Salivary gland	Serous acini	Glandular epithelium
Cluster023	26	19	Fundus	Fundic glands	Glandular epithelium
Cluster012	106	89	Fundus>antrum	Mucous neck/gastric glands	Glandular epithelium
Cluster018	61	51	Antrum	Pyloric glands	Glandular epithelium
Cluster031	20	17	Duodenum>fundus>intestine	Complement (crypts/goblet cells)	Glandular epithelium
Cluster014	79	60	Duodenum	Glandular/epithelial	Glandular epithelium
Cluster001	801	653	Ileum>jejunum	B cell/cell cycle	Immune
Cluster039	17	12	Intestine/stomach/salivary	Plasma B cell	Immune
Cluster020	37	30	High in intestine (small>large)/fundus	T cell	Immune
Cluster055	11	6	High in intestine (small>large)/fundus	T cell	Immune
Cluster027	22	16	Stomach/intestine-variable between animals	IFN response	Immune
Cluster033	18	15	Stomach/intestine-variable between animals	Immune response	Immune
Cluster037	17	15	General-higher in stomach/intestine	Immune-related	Immune
Cluster011	122	91	General-higher in stomach/intestine	MHC class 1 antigen presentation	Immune
Cluster022	32	30	General-higher in intestine/stomach	MHC class 2 antigen presentation (immune)	Immune
Cluster026	23	21	Pylorus	Neuronal	Neuronal
Cluster010	124	95	General-higher in pylorus>antrum	Neuronal	Neuronal
Cluster004	456	336	Tongue/upper oesophagus	Skeletal muscle	Muscle
Cluster002	532	382	Pylorus/antrum/oesophagus>>general	Smooth muscle/ECM (Fibroblast)	Muscle
Cluster052	12	9	Esophagus	Cartilage	Muscle
Cluster030	20	17	Tongue/esophagus>pylorus	Muscle-related	Muscle
Cluster009	130	93	General-higher in tongue/u-esophagus/antrum/pylorus	Muscle-smooth/skeletal	Muscle
Cluster007	149	142	General-higher in ileum, rectum	Cell cycle-related	Pathway
Cluster019	58	54	General-higher in small intestine	Histones	Pathway
Cluster017	63	53	General-higher in muscle	Oxidative phosphorylation	Pathway

GI, gastrointestinal.

In analyzing these data we have attempted to relate the clusters to the cell composition of the GI tact, based on the gene membership of clusters and their expression pattern. The different samples varied significantly in their muscle content, so some of the largest clusters contained muscle-specific genes. GI-cluster 4 was enriched for genes known to be expressed specifically in skeletal muscle and were highly expressed in the tongue and esophageal samples (Figure 5b). In contrast, the genes in GI-cluster 2 were highly expressed throughout the GI tract, peaking in the pylorus sample. The cluster contained not only genes associated with smooth muscle but also many extra-cellular matrix (ECM)-associated genes identified previously from mouse data [15,48]. Expression of these genes was shared with other mesenchymal lineages (fat, adipose, bone) and they formed a separate cluster in the whole atlas data. GI-cluster 9 sits between GI-clusters 2 and 4 and comprises a set of genes expressed in both muscle types. Another cluster in this region of the graph (GI-cluster 17) contained many of the genes associated with oxidative phosphorylation (as discussed above) with a number of interesting and plausible new additions to this pathway. Finally, GI-cluster 10 genes were highly-expressed in the pylorus sample. The cluster contained numerous neuron-associated genes and may derive from neuronal/supporting cells that make up the enteric plexus. Although the motile and hormonal activity of the GI tract is controlled by a complex nervous system, neurons actually represent only a small percentage of the cells that make up the organ. Hence, their expression signature would appear to be relatively weak compared with other cell types.

The GI tract is also a major immune organ. It represents one of the main battle grounds in an animal's defense against invading pathogens because of the large surface area, the nutrient rich luminal environment and the requirement for a thin lining permeable to nutrients. It is, therefore, unsurprising that the largest cluster of genes (GI-cluster 1) contained many genes associated with the immune system, their expression being two- to three-fold higher in the ileum than other regions. The lower small intestine is known to be associated with increased immune surveillance and the presence of Peyer's patches (specialized lymphoid follicles associated with sampling and presentation of luminal antigens). The cluster analysis did not separate the immune cell types which are largely co-located in the lamina propria and lymphoid aggregates. Included in GI-cluster 1 were genes encoding many of the protein components of the B cell receptor complex (*CD19*, *CD22*, *CD79A/B*, *CR2*) but also numerous genes identified in the full atlas analysis as being expressed specifically by T cells or macrophages. Also evident in this cluster were many of the core components of the cell cycle, for example cyclins, DNA polymerases, kinesins, and so on, again identified in the whole atlas as a discrete cluster (atlas cluster 14). The association of cell cycle genes with an immune signature is most likely due to the high level of lymphocyte proliferation [49], which increases the proportion of cells undergoing mitosis relative to the rest of the organ. In the neighborhood of the main GI immune cluster were smaller clusters of immune-associated genes that were expressed in a distinct but related manner, perhaps connected to regional immune specialization. GI-cluster 20 contains many of the components of the T cell receptor complex (*CD2*, *CD3D/E/G*, *CD8A*) which could be aligned with the distribution of intraepithelial lymphocytes. The analysis also detected a small, heavily expressed cluster of plasma B cell genes (GI-cluster 39, high expression in salivary gland, stomach and along the length of the small and large intestines) and two small clusters of immune response genes (GI-clusters 27 and 33) that varied significantly in their level of expression between animals. Other clusters were enriched for MHC class 1 (GI-cluster 11) and class 2 (GI-cluster 22) antigen presentation pathway genes.

Although the lamina propria of the gut contains the largest macrophage population in the body [50], many of the macrophage-specific genes identified in the whole atlas were not detectable in GI-cluster 1. For each of the genes in the macrophage cluster as defined in the full atlas dataset, we calculated the ratio of their highest expression in macrophages to their highest expression across GI tract samples. The average ratio was around 5, suggesting that macrophages provide around 20% of the total mRNA yield from the gut. The genes that were under-expressed based upon this ratio were derived mainly from atlas cluster 18, the subset of macrophage-expressed genes that was enriched in alveolar macrophages. The most repressed was *CYP7A1*, the cholesterol-7-hydroxylase, which metabolizes bile acids. The other striking feature was the large number of genes for C-type lectins, including *CLEC5A *(MDL1), *CLEC7A *(dectin), *CD68 *(macrosialin), *CLEC4D *(MCL), *SIGLEC1 *(sialoadhesin), *CLEC13D *(MCR1, CD206), *CLEC4E *(mincle) and *CLEC12B*, that are highly-expressed in alveolar macrophages but appeared down-regulated in the GI tract. This pattern indicates that macrophages of the gut are distinct from those of the lung and blood, perhaps adapted to be hypo-responsive to food-derived glycoproteins where those of the lung must use the same receptors to recognize and engulf potential pathogens. The phenotype of lamina propria macrophages may also vary within different regions of the GI tract thereby breaking up their expression signature.

The epithelial layer exhibits a great diversity between different GI compartments, its structure and function changing in line with requirements. Many clusters correlated with the known region-specific expression of structural proteins and solute carriers described above. GI-clusters 3 and 8, containing specific keratin genes, are related to the stratified squamous epithelial populations that protect against abrasion and mechanical damage to the underlying tissues in the tongue and esophagus. Genes in GI-cluster 3 tended to be expressed in equal levels in the tongue and lower esophagus, whereas genes in GI-cluster 8 were more restricted in their expression to the tongue. These genes define the specific signature of stratified squamous epithelial populations present in this organ. Similarly GI-clusters 13 and 16 which were high in the salivary gland or along the entire length of the gut, respectively, likely represent genes specifically expressed in the stratified or ciliated columnar epithelium present in these organs. Among the columnar epithelium populations, which line the gut from the stomach to the rectum, there was region-specific differentiation, reflected by the differing levels of expression of genes along the longitudinal axis of the intestine and the presence of specific populations of glandular cells. Enriched in GI-cluster 5 were many transcripts (representing 251 unique gene IDs) that were expressed specifically in the small intestine and encode the machinery for the digestion and absorption of nutrients. In contrast, there were relatively few genes expressed specifically in the colon (GI-clusters 25 and 29, representing 37 unique gene IDs) and little evidence of functional compartmentalization of expression along that organ. Among these genes many matched the known markers of this tissue but others were novel. There are various glandular and endocrine cell populations that are integral to the columnar epithelial lining and in many cases have their origins in the same epithelial stem cell populations located at the base of the crypts. Because they inhabit specific niches within the GI tract, genes expressed specifically within them have a unique expression pattern. For this reason, we can assign the genes in GI-cluster 23 with some confidence to expression in the fundic glands, GI-cluster 18 genes to pyloric glands and GI-cluster 12 genes to mucous secreting superficial gastric glands. These assignments are also strongly supported by the gene membership of these clusters and the lists expand the complement of genes known to be expressed in these specialized glandular systems. The genes in GI-cluster 14 were likely expressed in glandular/endocrine cells present only in the duodenum. Finally, genes expressed in the salivary gland could be segregated to those expressed in serosal (GI-cluster 6) or mucosal (GI-cluster 15) acini. While both were exclusively expressed in the salivary gland they separate the two salivary gland samples, presumably due to chance sampling of different regions of the gland.

**Figure 6:**
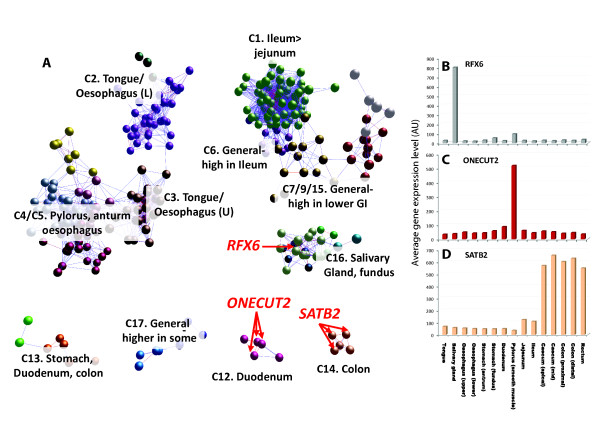
**GI tract transcription factor network**. A plot of the relationships in expression among the complement of transcription factors (TFs) expressed in the porcine GI tract. TFs with similar expression profiles group together and groupings likely represent regulatory units that work together to control cellular differentiation within regions of the organ. A number of TFs have been selected that are expressed in a very region-specific manner but whose known biology has not previously been associated with a functional role within this organ. GI, gastrointestinal.

In our previous analysis of a mouse cell atlas, specific clusters frequently contained the transcription factors that regulated them, and their promoters were over-represented with the motifs that are the targets of those factors [32]. We analyzed a set of candidate transcription factors (TFs) encoded by the human genome [51] as a correlation network (r >0.8, MCL2.2 Figure 6). Clusters of TFs that had a preference in their expression for one or multiple regions of the GI tract grouped together. The expression patterns of numerous other TFs imply previously unrecognized roles in regulating cell differentiation in this organ. RFX6 is classically associated with regulating insulin expression and has recently been shown to be essential for islet cell differentiation in the murine pancreas [52,53]. In the pig GI tract, the *RFX6 *gene was highly expressed in the salivary gland, with significant expression in the duodenum (Figure 6b). We suggest that the RFX6 protein could also contribute to epithelial/endocrine differentiation in these organs. This suggestion is supported by protein expression data [54], and the discovery that mutations in this gene in human Mitchell-Riley syndrome are associated with duodenal and jejunal atresia [52]. The ONECUT2 protein is a member of a small TF family that contains a cut domain and an atypical homeodomain. ONECUT2 has been associated with the regulation of retinal development [55] and pancreatic and enteric endocrine differentiation [56]. In the pig gut, the gene was highly and specifically expressed in the duodenum (Figure 6c) and was tightly coexpressed with the TF *PDX1 *(Pancreatic and duodenal homeobox 1), a gene which is expressed by duodenal enterocytes [54], suggesting a role in defining epithelial differentiation in the region of the intestine. Finally, SATB2 is a homeobox protein with known roles in osteoblast [57,58] and neuronal [59,60] differentiation. The recently characterized HSA2q33.1 microdeletion syndrome is associated with genomic deletion of all or part of the human *SATB2 *gene [61]. In the pig, expression of this gene was exclusively found in the lower bowel, consistent with human protein expression data [54] and its utility as a marker of colorectal derived cancers [62]. This specific expression in the epithelium of the large intestine would predict a defining role in this region.

## Conclusions

This work describes the first detailed analysis of the transcriptional landscape of the pig. Since the pig is a large animal with a physiology that is closer to man's than is that of mouse, this analysis provides a major new resource for understanding gene expression with respect to the known physiology of mammalian tissues and cells. At the single gene level, this dataset represents a comprehensive survey of gene expression across a large range of pig tissues. In instances where the expression of a gene is regulated in a tissue-specific manner it represents a good starting point for understanding its likely cellular expression pattern and, therefore, its functional role. The availability of the data on the BioGPS web portal renders the data amenable to such queries. However, it is the ability to understand the expression of a gene in the context of others that makes this analysis unique. Correlation analysis and the use of advanced network visualization and clustering techniques go beyond standard pairwise hierarchical approaches in defining coexpression relationships between genes. The approach used here allows us to capture and visualize the complexity of these relationships in high dimensional data, rendering large proportions of the data available for analysis. Using this network clustering approach we have been able to recapitulate known expression and functional relationships between genes as well as infer new ones based on guilt-by-association. The detailed analysis of the transcriptional landscape of the gastrointestinal tract provides the first comprehensive view of the regional specialization of this organ in a large animal, and has highlighted numerous candidate genes that may underlie genetic diseases of the human gastrointestinal tract such as colitis and cancer.

## Methods

### Design of the 'Snowball' array and annotation of the probesets

Porcine expressed sequences (cDNA) were collated from public data repositories (ENSEMBL, RefSeq, Unigene and the Iowa State University ANEXdb database) to create a non-overlapping set of reference sequences. A series of sequential BLASTN analyses, using the National Center for Biotechnology Information (NCBI) blastall executable, were performed with the -m8 option. The initial subject database comprised 2,012 sequences of manually annotated *S. scrofa *gene models from Havana provided by Jane Loveland (The Sanger Institute) on 29 July 2010, plus 21,021 sequences acquired using Ensembl BioMart *Sscrofa *(build 9, version 59 on 22 July 2010). For each iteration, query sequences that did not have an alignment with a bitscore in excess of 50 were added to the subject database prior to the next iteration.

The iterations involved the following query datasets:

1. 35,171 pig mRNA sequences from NCBI, downloaded on 15 July 2010: 6,286 added to subject database

2. 7,882 pig RefSeq sequences from NCBI, downloaded on 15 July 2010: 0 added to subject database (all RefSeq's were already represented in source 1)

3. 43,179 pig Unigene sequences from NCBI, downloaded on 15 July 2010 (filtered to include only those longer than 500 bases): 10,125 added to subject database

4. 121,991 contig sequences, downloaded from Iowa Porcine Assembly v1 (http://www.anexdb.orgt) on 30 July 2010 (filtered to include only those longer than 500 bases): 10,536 added to subject database.

5. 2,370 miRNA sequences (pig, cow, human, mouse), downloaded from miRbase, 30 July 2010 (Release 15, April 2010, 14197 entries): all added without BLASTN analysis.

The final subject database comprised 52,355 expressed sequences.

To facilitate the design of array probes that were uniformly distributed along the entire length of transcripts, transcripts were split into several probe selection regions (PSRs), each of which was then the target for probe selection. The size of each PSR, typically around 150 nucleotides, was determined by the length of the input sequence, with the ultimate aim being to obtain 20 to 25 probes per transcript. Oligonucleotide design against the approximately 343,000 PSRs was performed by Affymetrix (High Wycombe, UK). In addition, standard Affymetrix controls for hybridization, labelling efficiency and non-specific binding were included on the array (a total of 123 probesets) together with complete tiling probesets for 35 porcine-related virus genome sequences (both strands, center-to-center gap of 17 nucleotides) for possible future infection-based studies. The final array is comprised of 1,091,987 probes (47,845 probesets) with a mean coverage of 22 probes/transcript.

Initial annotation of the gene models was obtained from the sequence sources and converted into an annotation set using the AnnotateDbi Bioconductor package. However, following this exercise many probesets were without useful annotation. Therefore, the original sequences from which the probes had been designed were blasted against NCBI Refseq in order to impute the most likely orthologous gene of the 'unannotated' pig transcripts. In order to have one gene per query sequence the following annotation pipeline was followed:

1. For each query the hit with lowest e-value within each species was chosen.

2. Genes with e-value hits <1e-9 against *Homo sapiens *were annotated with HUGO (Human Genome Organization) Gene Nomenclature Committee (HGNC) names/descriptions; however, genes with matches starting with 'LOC' were not used.

3. Step 2 was repeated using in order: S. scrofa, Bos taurus, Pan troglodytes, Mus musculus, Canis lupus familiaris, Pongo abelii, Equus caballus, Rattus norvegicus, Macaca mulatta.

4. Step 3 was repeated using any other species (in no particular order) to which a hit could be obtained.

5. For the remaining probes LOC gene annotations were used from (in order of priority): *H. sapiens, S. scrofa, B. taurus, P. troglodytes, M. musculus*

6. Everything else was used, in no particular order.

Out of 47,845 sequences represented on the array, 27,322 probesets have annotations that correspond to a current (15 December 2011) HGNC symbol for human protein coding gene, 14,426 of which are unique (out of a total 19,219 listed by HGNC). The remaining probesets were annotated with the information available for those sequences. The array design has been submitted to ArrayExpress (AcNo. A-AFFY-189).

### Tissues and cells

The majority of fresh tissue samples were obtained from young Landrace pigs (one male, three female 12- to 16-weeks old) that were being sacrificed for another study examining normal expression patterns in hematopoietic cell lineages. Pigs were sedated with ketamine (6 mg/kg) and azaperone (1 mg/kg), left undisturbed for a minimum of 15 minutes, and then killed by captive bolt. Tissues were dissected and a small piece immediately snap-frozen on dry ice and stored in a -155°C freezer until RNA extraction. All tissues were collected within a window of 10 to 90 minutes following the death of the animal. Samples of adult testis (Large White-Landrace-Duroc cross, eight- years-old) and placenta (Large White-Landrace cross, gestation day 50) that were not obtainable from the young animals were collected separately. Samples of blood and three different macrophage populations were also obtained from other animals. Blood samples were collected by jugular venepuncture of 8- to 12-week old Landrace males and 3 ml was placed in Vacuette Tempus Blood RNA tubes (Applied Biosystems, Warrington, UK) and stored at 4°C until RNA extraction. Alveolar macrophages were collected from the same animals by washing the left caudal/diaphramatic lung lobe with PBS (using 200 to 250 ml) followed by centrifugation of the bronchoalveolar lavage fluid at 800 g for 10 minutes; the supernatant (alveolar wash fluid) was retained. The alveolar macrophages were washed once with PBS prior to analysis. Bone marrow- (BMDM) and monocyte-derived macrophages (MDM) were generated from primary monocytes. A total of 400 ml of blood was collected together with five posterior ribs from each side of male Large White-Landrace pigs of 8- to 12-weeks of age. The buffy coat (after spinning the blood for 15 minutes at 1200 g) was mixed to one volume of RPMI and separated on a Ficoll gradient (Lymphoprep, Axis-Shield, Norway) for 25 minutes at 1,200 g. Peripheral blood mononuclear cells (PBMC) were then washed twice (10 minutes at 600 g, then 10 minutes at 400 g) with PBS. Bone-marrow cells (BMC) were isolated and cryopreserved at -155°C as previously described [33]. Both BMC and PBMC were thawed and derived into macrophages in the presence of recombinant human CSF-1 for five to seven days. BMDM and MDM were then treated with LPS from *Salmonella enterica *serotype Minnesota Re 595 (L9764, Sigma-Aldrich, Saint-Louis, USA) at a final concentration of 100 ng/ml and RNA was collected at 0 and 7 hours.

Total RNA was extracted using the RNeasy kit as specified by the manufacturer (Qiagen Ltd, Crawley, UK). RNA concentration was measured using ND-1000 Nanodrop (Thermo Scientific, Wilmington, USA). The quality was assessed by running the samples on the RNA 6000 LabChip kit (Agilent Technologies, Waldbronn, Germany) with the Agilent 2100 bioanalyzer. A total of 500 ng of total RNA was amplified using the Ambion WT Expression Kit (Affymetrix). A total of 5.5 µg of the resulting cDNA was fragmented and labelled using the Affymetrix Terminal Labelling Kit. The fragmented and biotin labelled cDNA was hybridized to the Snowball arrays, using the Affymetrix HybWashStain Kit and Affymetrix standard protocols. The fluidics protocol used was FS_0001. In total, 111 arrays were run on samples derived from 65 tissue/cell types.

All animal care and experimentation was conducted in accordance with guidelines of The Roslin Institute and the University of Edinburgh and under the Home Office project licence number PPL 60/4259.

### Data quality control and analysis

The quality of the raw data was analyzed using the arrayQualityMetrics package in Bioconductor (http://www.bioconductor.org/) and scored on the basis of five metrics, namely maplot, spatial, boxplot, heatmap and rle in order to identify poor quality data [63]. Arrays failing on more than two metrics, were generally removed. However, in a number of cases after examining the data, particularly from a number of the macrophage samples, it was considered that their poor quality control (QC) score was down to the samples being significantly different from the others but not of poor quality. RNA samples from the pancreas were partially degraded and consequently these data were scored as being of a lower quality, but were left in the final analysis due to yielding a cluster of pancreatic marker genes. A further QC step involved the creation of a sample-sample correlation network where edges represented the Pearson correlation value and nodes the samples [see Additional file 10, Figure S3]. In a number of cases samples clearly did not group with similar samples, indicating a likely error at the point of collection or during processing and these samples were removed from the analysis. Details of the tissues/cells used in this study are given in Additional file 1, Table S1.

Following QC, data from 104 arrays run on samples derived from 62 tissue/cell types were normalized using the robust multi-array average (RMA) expression measure [64]. In order to make these data accessible all raw and normalized data have been placed in ArrayExpress (AcNo. E-MTAB-1183) and the expression and graph layout files have been made available to support future graph-based analyses using BioLayout *Express*^3D ^[see Additional files 2 and 3]. Furthermore, the data have been uploaded onto the BioGPS web site (http://biogps.org) [65] enabling the search for a profile of an individual gene and those correlated with it. This site also supports mouse and human atlas datasets allowing the direct comparison of gene expression profiles across species. Following data normalization, samples were ordered according to tissue type and the dataset was saved as an '.expression' file and then loaded into the network analysis tool BioLayout *Express*^3D ^[30], as described previously [31]. A pairwise Pearson correlation matrix was calculated for each probeset on the array as a measure of similarity between the signal derived from different probesets. All Pearson correlations with r ≥0.7 were saved to a '.pearson' file and a correlation cut off of r = 0.8 was used to construct a graph containing 20,355 nodes (probesets) and 1,251,575 edges (correlations between nodes above the threshold). The minimum sub-graph component size included in the network was five. Graph layout was performed using a modified Fruchterman-Rheingold algorithm [66] in three-dimensional space in which nodes representing genes/transcripts are connected by weighted, undirected edges representing correlations above the selected threshold. Gene coexpression clusters were determined using the MCL algorithm [67], which has been demonstrated to be one of the most effective graph-based clustering algorithms available [68]. An MCL inflation value of 2.2 was used as the basis of determining the granularity of clustering, as it has been shown to be optimal when working with highly structured expression graphs [30]. Clusters were named according to their relative size, the largest cluster being designated Cluster 1. Graphs of each dataset were explored extensively in order to understand the significance of the gene clusters and their relevance to the cell biology of pig tissues. A cluster was annotated if the genes within it indicated a known function shared by multiple members of the cluster. These analyses were supplemented by comparison of the clusters with tissue- and cell-specific clusters derived from network-based analyses of a human tissue atlas and an atlas of purified mouse cell populations [14,32] and tissues, Gene Ontology [69], The Human Protein Atlas database [70] and comprehensive reviews of the literature (data not shown). A description of the average profile and gene content of the major clusters can be found in Additional file 4, Table S2.

In order to focus down specifically on expression patterns along the porcine GI tract, the data from these tissues (30 samples in total) were treated separately. Due to the smaller size of this dataset there is a greater chance of low intensity data being correlated by chance, so data were removed for all probesets where the maximum normalized expression value never exceeded a value of 50 in any of the GI samples. This filtering left 29,918 probesets. These data were then subjected to network analysis at a correlation cut-off value of r = 0.90 and clustered using an MCL inflation value of 2.2. This network was inspected manually and clusters were removed where they showed no particular region-specific expression pattern or were most likely formed due to contamination of GI tissues with surrounding tissues (for example, it would appear that one of the rectal samples was contaminated with glandular tissue of the reproductive tract). The remaining data were again subjected to network analysis (r = 0.90) producing a graph composed of 5,199 nodes/195,272 edges [see Additional file 6, Figure S2] which was clustered using an MCL inflation value of 1.7 (the lower inflation value reducing the overall number of clusters). The resulting cluster analysis of 120 clusters with a membership between 801 and 5 probesets, was then explored in order to annotate the most likely cellular source of the expression signatures observed. This was aided by reference to the cluster analysis of the whole dataset.

## Abbreviations

BMC: bone marrow cells; BMDM: bone marrow-derived macrophages; CNS: central nervous system; ECM: extra-cellular matrix; GI: gastrointestinal; HGNC: HUGO (Human Genome Organization) Gene Nomenclature Committee; LOC: LocusLink; LPS: lipopolysaccharide; MCL: Markov cluster algorithim; MDM: monocyte-derived macrophages; ncRNAs: non-coding RNAs; PBMC: peripheral blood mononuclear cells; PBS: phosphate-buffered saline; PSRs: probe selection regions; RMA: robust multi-array average; RNAseq: sequencing of RNA; snoRNAs: small nucleolar RNAs; TCA: tricarboxylic acid; TFs: transcription factors.

## Competing interests

The authors declare that they have no competing interests.

## Authors' contributions

TCF helped conceive the idea, was instrumental in organizing the design of the Snowball array, led the dissection of tissues and generation of the primary data, performed the network analysis of the data and was the primary author of the paper. AI was primarily responsible for working up the pig sequence resource and worked with Affymetrix in organizing the design and first-pass annotation of the array. He was also responsible for the primary data quality control and normalization. DB was responsible for the reannotation of the array above and beyond simply matching the known pig genes to reference databases. JKB aided in the design of the experiment, led the dissection of the CNS and other peripheral tissues and brought a clinician's perspective to anatomical dissection. RK and AT helped in the collection of tissues and were responsible for generating the macrophage samples. MB, DD, LF and RA helped in the collection of tissues. AD was responsible for running the arrays and in the work up of the primary data. SR assisted with the work up of the data and its distribution on the website http://www.macrophages.com. KMS helped conceive the work and was involved in collection of samples and writing the paper. CKT provided access to the Iowa State University database of expressed pig sequences (http://www.anexdb.org). AIS and CW were instrumental in loading the data on to BioGPS. ALA and DAH helped conceive the work, suggested inputs for the array's design and were primary authors of the paper. All authors read and approved the final manuscript.

## Supplementary Material

Additional file 1 **Table S1. Details of the tissues and cells used for this study. List and details of tissues and cells used for this study**. Click here for file

Additional file 2 **Pig atlas '.expression' file**. File of all RMA normalized data describing the expression pattern of the majority of porcine genes across 63 tissue/cell types. File may be opened in Microsoft Excel or BioLayout *Express*^3D^. Click here for file

Additional file 3 **Pig atlas network '.layout' file**. Precalculated network layout of the graph used in this analysis. The network contains 20,355 nodes (probesets) and 1,251,575 edges (correlations ≥0.8) that can be visualized in BioLayout *Express*^3D ^(http://www.biolayout.org). This is a large graph and requires good hardware (a decent graphics card and sufficient RAM) to render and navigate. See http://www.biolayout.org/download/requirements/ for the requirements to run this program. Click here for file

Additional file 4 **Table S2. Fully annotated cluster analysis (r > 0.8, MCL inflation 2.2) of the pig expression atlas network**. The gene/transcript membership of each cluster is defined together with a description of the average expression profile of each of the major clusters and known association of the genes with a tissue, cell type or pathway. Click here for file

Additional file 5 **Figure S1. Expression profiles of selected gene family members/functionally related genes along the length of the GI tract**. A number of gene families were selected and the profile of specific members investigated. **A**. The keratins are a large gene family where the expression of individual members is associated with specific classes of epithelial/dermal layers. In this case there are numerous family members expressed in the stratified squamous epithelia of the tongue and esophagus whereas others are expressed specifically in columnar epithelia of the mid to lower GI tract. **B**. Expression of digestive enzymes is in most cases restricted to the small intestinal enterocytes but individual patterns of expression along the longitudinal axis of the region do vary in line with requirements. **C**. In common with the genes shown in B, expression of the solute transporters associated with absorption mirrors the requirement for their activity being expressed in a region-specific manner along the small and large intestine. **D**. Mucins play a crucial role in the lubrication and protection of the GI tract. The profiles of a number of gene family members are shown, some of which are highly expressed in the salivary gland (*MUC12, MUC19*), others in the stomach (*MUC1, MUC5AC*) and *MUC4*'s expression is restricted to the colon. **E**. Regulating many aspects of GI function are a range of hormones expressed by endocrine cells that line the organ. The expression of the hormone genes shown here is largely restricted to the stomach and duodenum. **F**. Expression of T and B cell marker genes whose expression peaks in the ileum where the immune cell content of the organ is at its highest. **G**. Myosins are essential components of muscle fibers and are utilized differently in different types of muscle. In this case they segregate according to the distribution of skeletal muscle (tongue, esophagus) or smooth muscle (other regions). **H**. Many collagens are required for the formation of the extracellular matrix that is a major component of connective tissues and is produced by various mesenchymal cells types particularly fibroblasts. These genes are consequently observed to be expressed along the entire GI tract albeit in a region-dependent manner. Click here for file

Additional file 6 **Figure S2. *Screenshots of the GI tract transcriptional network in 2D and 3D***. Visualization of the transcriptional network associated with the pig GI tract. The network contains 5,199 nodes (probesets) connected by 195,272 edges (transcript-to-transcript correlations above 0.9); node color represents cluster membership. The same graph rendered in a two dimensional plane (inset). Click here for file

Additional file 7 **Pig GI tract '.expression' file**. File of all RMA normalized data describing the expression pattern of a subset of porcine genes across 15 regions of the porcine GI tract. File may be opened in Microsoft Excel or BioLayout *Express*^3D^. Click here for file

Additional file 8 **Pig GI tract network '.layout' file**. Precalculated network layout of the graph used in this analysis. The network contains 5,199 nodes (probesets) 195,272 edges (correlations ≥0.9) that can be visualized in BioLayout *Express*^3D^. Click here for file

Additional file 9 **Table S3. Fully annotated cluster analysis (r > 0.9, MCL inflation 1.7) of the pig gastrointestinal tract expression network**. The gene/transcript membership of each cluster is defined together with a description of the average expression profile of each of the major clusters and known association of the genes with a tissue, cell type or pathway. Click here for file

Additional file 10 **Figure S3. Sample-to-sample Pearson correlation graph. A sample-to-sample Pearson correlation was calculated and relationships r > 0.91 used to group samples together**. Nodes represent different samples and the edges relationships above the cutoff, the thicker/redder the line the greater the similarity between samples. The graph has been clustered using an MCL inflation value of 6. Click here for file
