# Supplementary figures and images for: A gene expression atlas of the domestic pig
Source: BMC Biol. 2012 Nov 15;10:90. doi: 10.1186/1741-7007-10-90 (PMC3814290; doi:10.1186/1741-7007-10-90)

## Expression Profile of Marker Genes along the Pig GI Tract

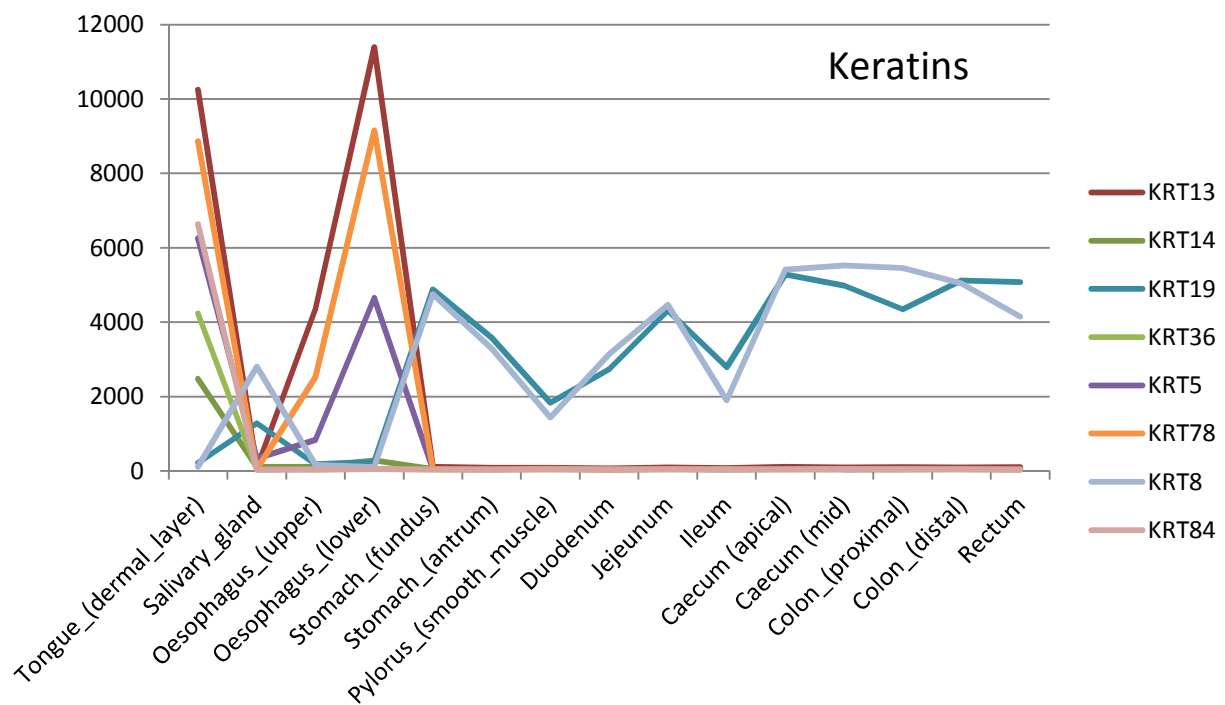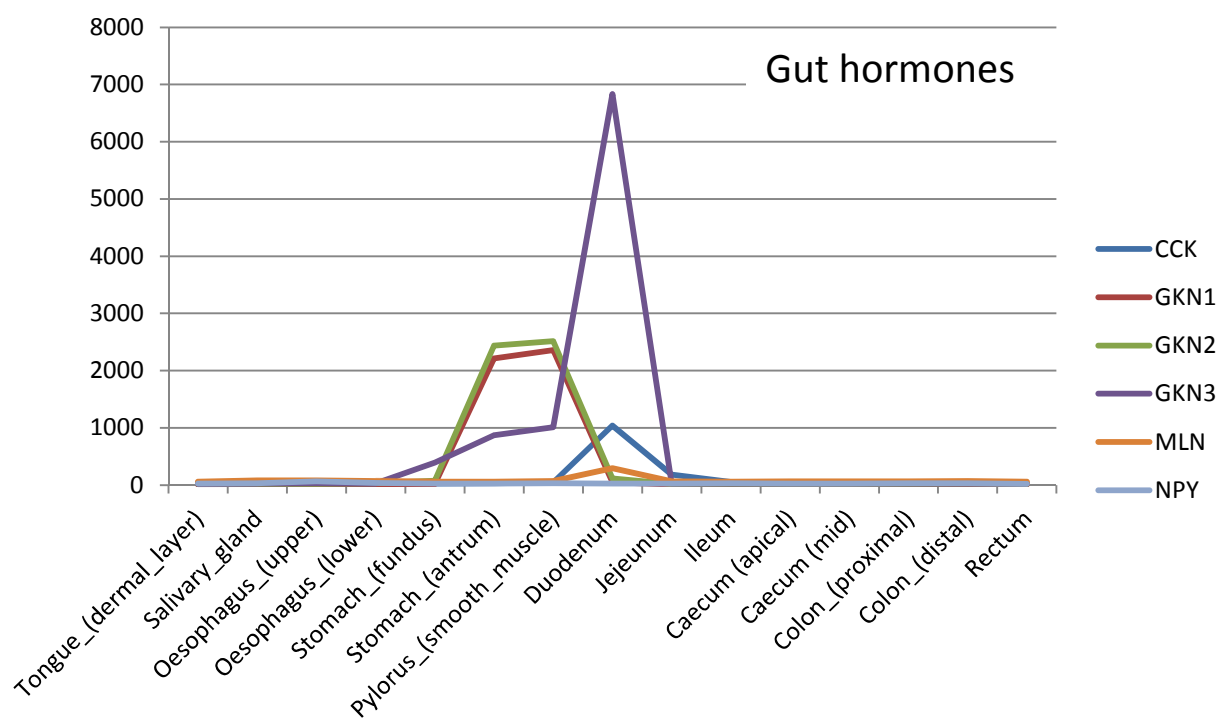

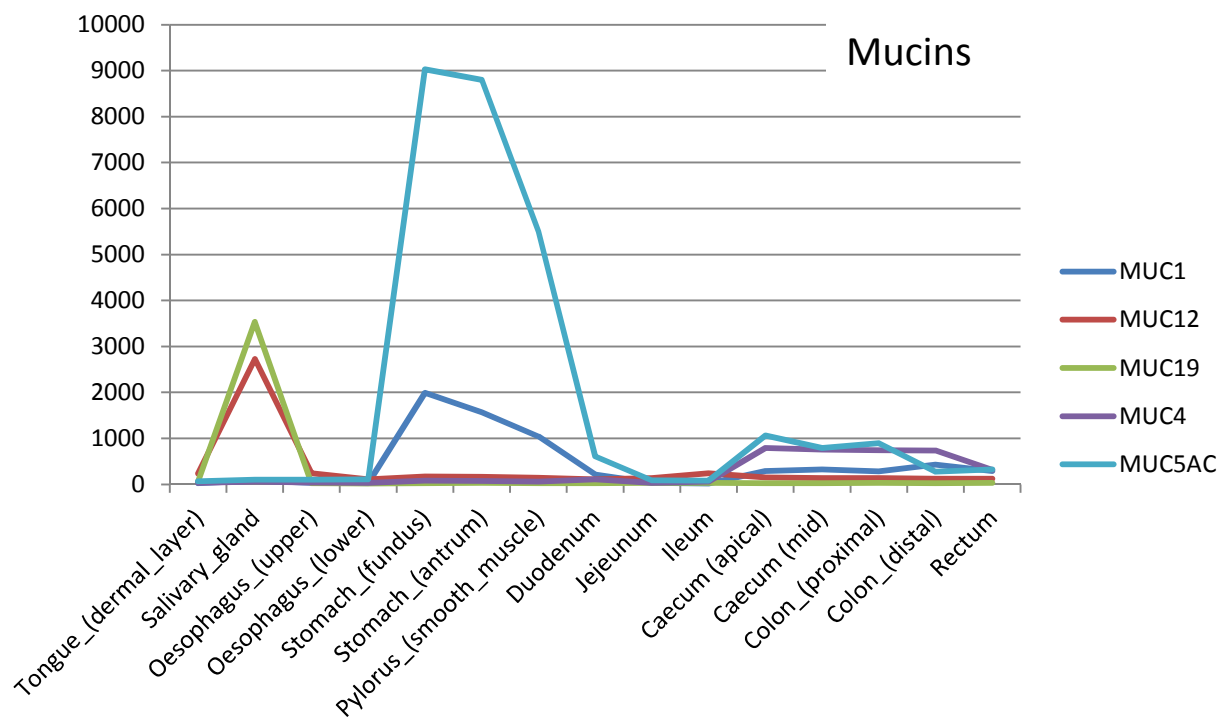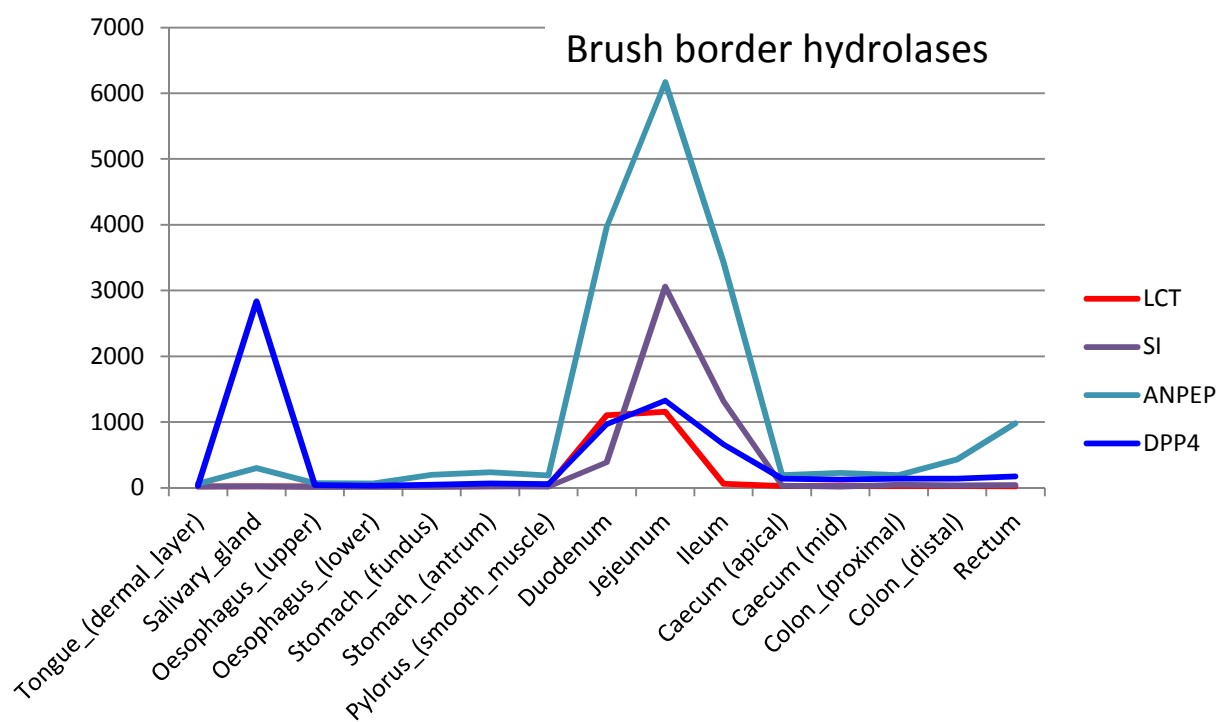

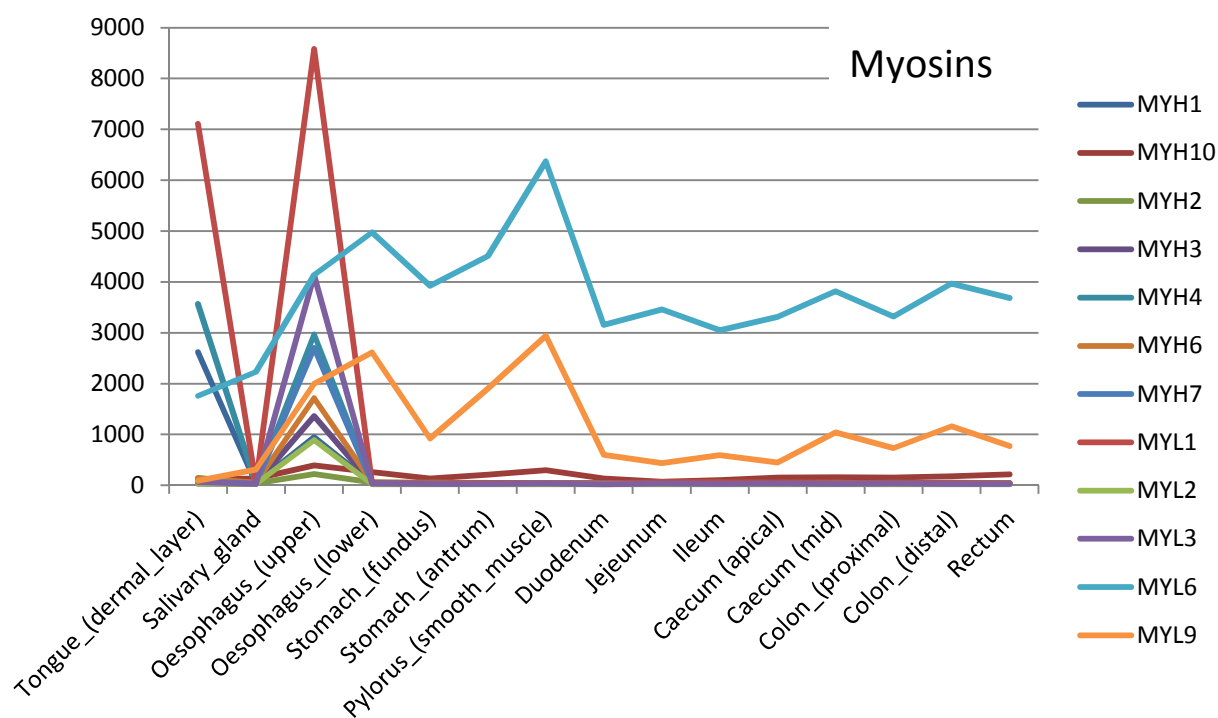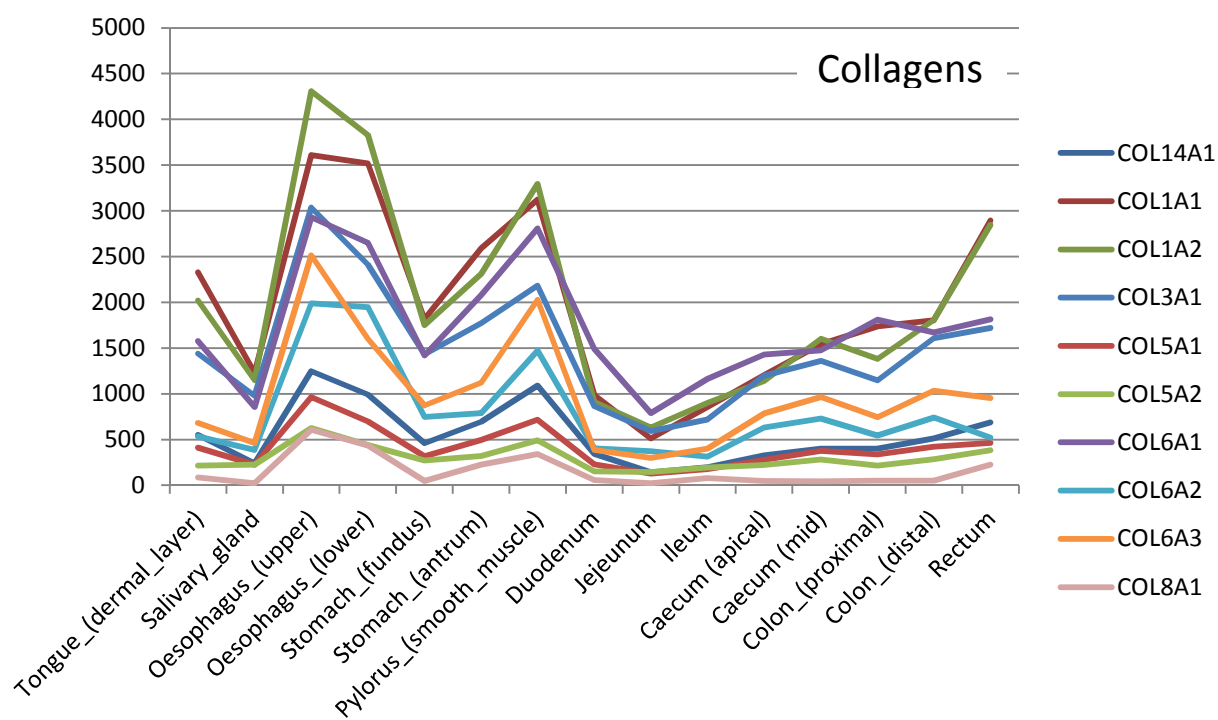

Supplement: Additional file 5 — Figure S1. Expression profiles of selected gene family members/functionally related genes along the length of the GI tract. A number of gene families were selected and the profile of specific members investigated. A. The keratins are a large gene family where the expression of individual members is associated with specific classes of epithelial/dermal layers. In this case there are numerous family members expressed in the stratified squamous epithelia of the tongue and esophagus whereas others are expressed specifically in columnar epithelia of the mid to lower GI tract. B. Expression of digestive enzymes is in most cases restricted to the small intestinal enterocytes but individual patterns of expression along the longitudinal axis of the region do vary in line with requirements. C. In common with the genes shown in B, expression of the solute transporters associated with absorption mirrors the requirement for their activity being expressed in a region-specific manner along the small and large intestine. D. Mucins play a crucial role in the lubrication and protection of the GI tract. The profiles of a number of gene family members are shown, some of which are highly expressed in the salivary gland (MUC12, MUC19), others in the stomach (MUC1, MUC5AC) and MUC4's expression is restricted to the colon. E. Regulating many aspects of GI function are a range of hormones expressed by endocrine cells that line the organ. The expression of the hormone genes shown here is largely restricted to the stomach and duodenum. F. Expression of T and B cell marker genes whose expression peaks in the ileum where the immune cell content of the organ is at its highest. G. Myosins are essential components of muscle fibers and are utilized differently in different types of muscle. In this case they segregate according to the distribution of skeletal muscle (tongue, esophagus) or smooth muscle (other regions). H. Many collagens are required for the formation of the extracellular matrix that [file 1741-7007-10-90-S5.PDF]

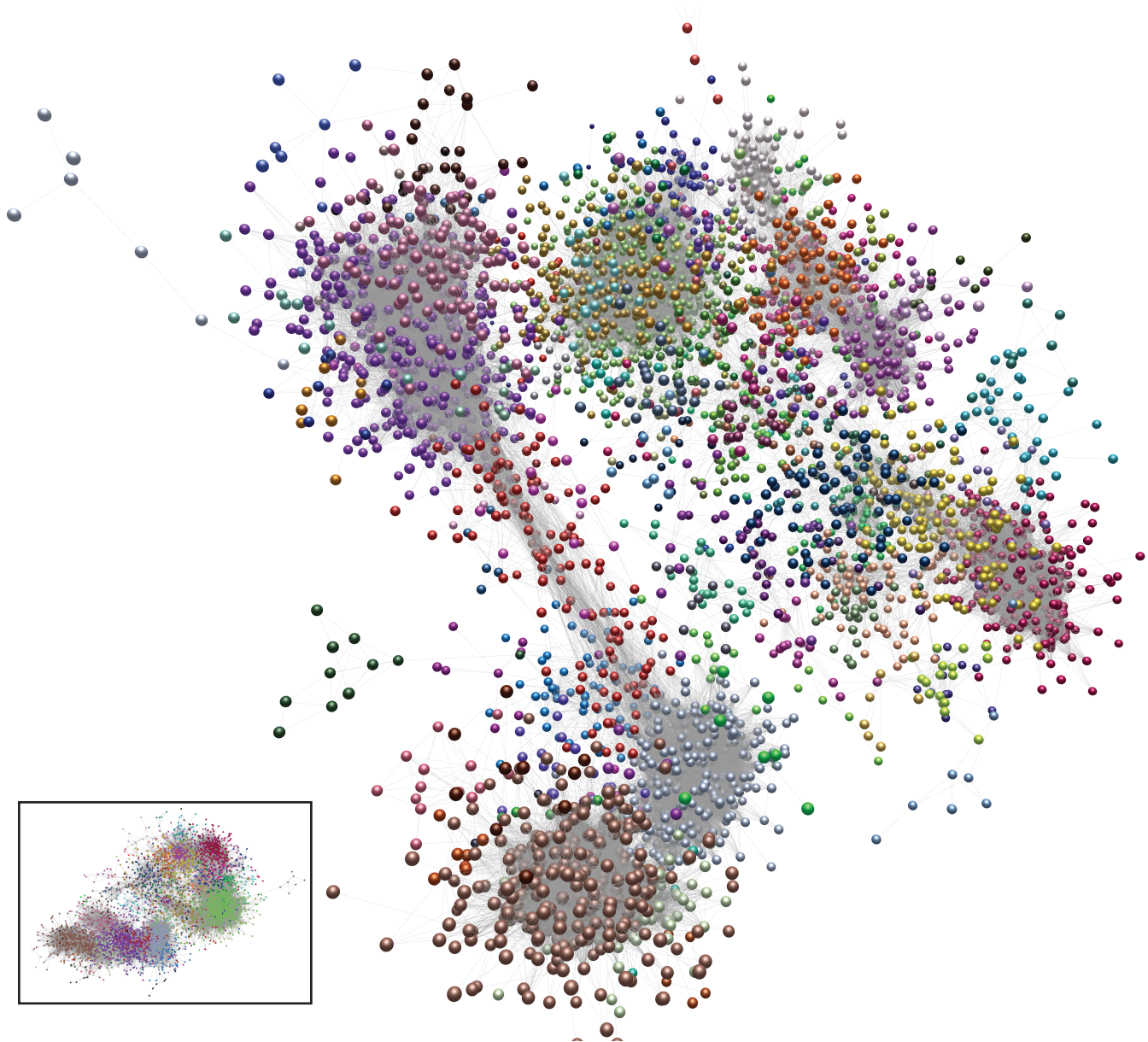

Supplement: Additional file 6 — Figure S2. Screenshots of the GI tract transcriptional network in 2D and 3D. Visualization of the transcriptional network associated with the pig GI tract. The network contains 5,199 nodes (probesets) connected by 195,272 edges (transcript-to-transcript correlations above 0.9); node color represents cluster membership. The same graph rendered in a two dimensional plane (inset). [file 1741-7007-10-90-S6.PDF]

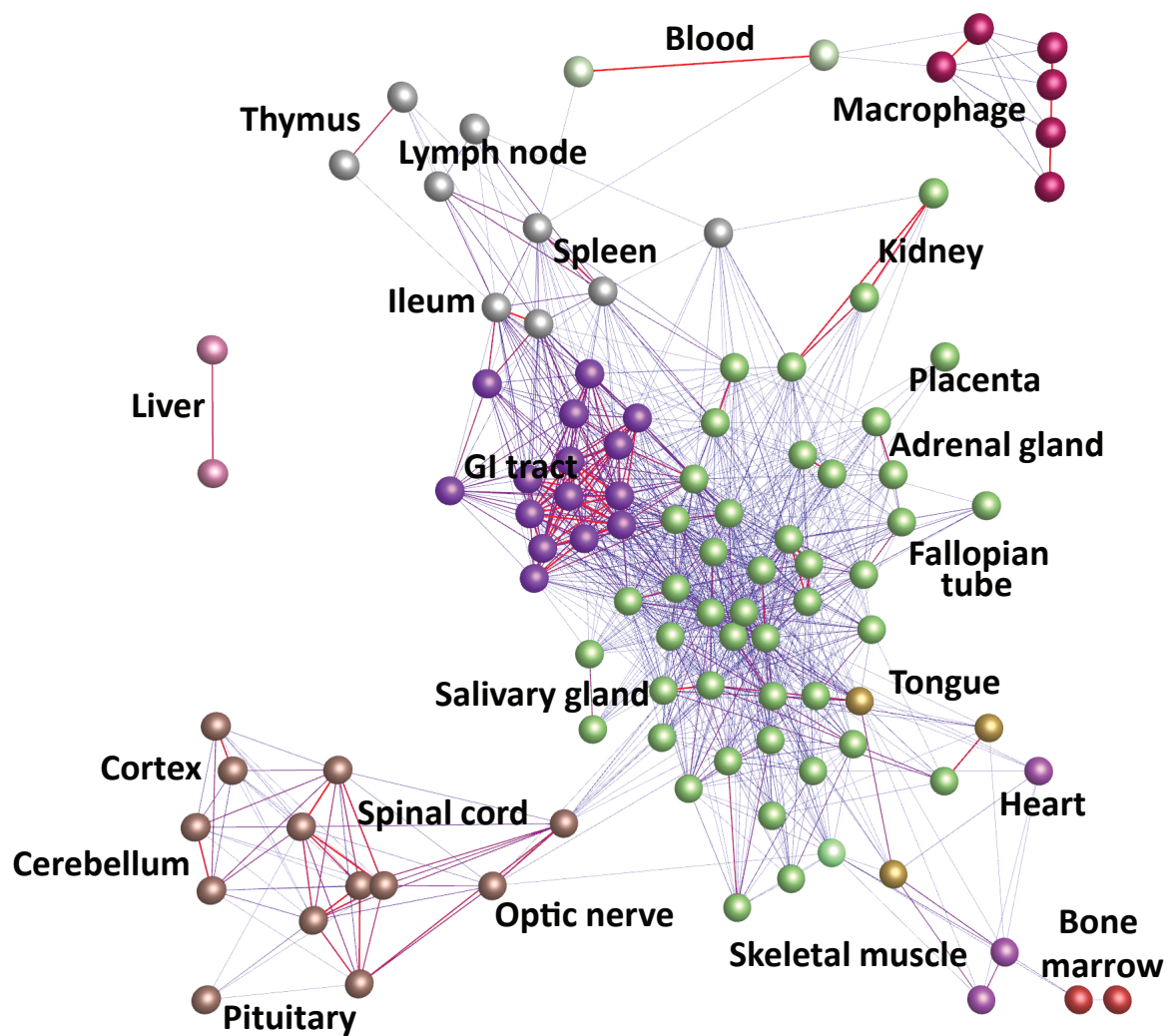

Supplement: Additional file 10 — Figure S3. Sample-to-sample Pearson correlation graph. A sample-to-sample Pearson correlation was calculated and relationships r > 0.91 used to group samples together. Nodes represent different samples and the edges relationships above the cutoff, the thicker/redder the line the greater the similarity between samples. The graph has been clustered using an MCL inflation value of 6. [file 1741-7007-10-90-S10.PDF]
